# Variation of the Thread Thickness After Thread Lifting: An Ultrasonographic Evaluation

**DOI:** 10.1111/jocd.70250

**Published:** 2025-06-24

**Authors:** Hüray Hügül, Kadir Atacan Yıldız, Rozerin Neval Altunkalem, Züleyha Aydın, Zekayi Kutlubay

**Affiliations:** ^1^ Dermatology Hüray Hügül Private Clinic Antalya Turkey; ^2^ Department of Dermatology, Cerrahpaşa Medical Faculty İstanbul University‐Cerrahpaşa İstanbul Turkey; ^3^ Department of Dermatology Kahta State Hospital Adıyaman Turkey; ^4^ Department of Biostatistics, Cerrahpaşa Medical Faculty İstanbul University‐Cerrahpaşa İstanbul Turkey

**Keywords:** long‐term, silicone thread, thickness, thread lift, ultrasonography

## Abstract

**Background:**

Thread lifting for the management of skin sagging is a well‐established method of noninvasive facial rejuvenation; however, the literature regarding follow‐up with ultrasound imaging is sparse.

**Aims:**

The aim of this study was to assess the long‐term stability of nonabsorbable silicone threads by monitoring any changes in their thickness over time. Using ultrasound imaging, we tracked the threads' dimensional changes in patients to determine whether the threads maintain their structural integrity during the follow‐up period.

**Materials and Methods:**

A retrospective analysis was conducted on 51 patients who underwent thread lifting with silicone threads. Ultrasonography was used at follow‐up points to assess various parameters. These included the visibility of the threads, any changes in their thickness over time, potential migration of the threads in relation to the cannula entry point, as well as variations in echogenicity, which could suggest alterations in the thread material or surrounding tissue, along with any associated complications.

**Results:**

The patients who had their follow‐up ultrasound done 6 months or less after the procedure had a lower mean thread thickness compared to those who were evaluated after 6 months. After 6 months, however, the thread thickness remains within a fixed range. The most common adverse effect was swelling. There was no migration in any of the patients.

**Conclusions:**

Thread lifting is a procedure with long‐term durability. Ultrasound imaging can be utilized as a tool for follow‐up.

## 1 Introduction

The face has a unique arrangement of skin, fat, muscles, ligaments, and bones. Facial aging is a multifaceted process occurring as a result of various changes in these structures and in their relationship to each other. The growing demand for minimally invasive aesthetic enhancement has driven the advancement of different techniques used in facial rejuvenation, including the variety of threads used to counteract the effect of gravity [1, 2, 3].

Threads can be made of absorbable and nonabsorbable material. Although nonabsorbable threads have a long‐lasting effect, absorbable threads made of polydioxanone (PDO), poly‐L‐lactic acid (PLLA), polyglycolic acid (PGA), and polycaprolactone (PCL) may cause fewer complications. The most used absorbable thread type is PDO, and it contains hooks facing in opposite directions. Once the threads are placed under the skin, they are absorbed within 3 months and replaced by collagen bands that remain in place for 18 months [4, 5, 6, 7, 8].

Two types of nonabsorbable silicone threads have been used for the participants of this study: Infinite Thread and Spring Thread. The central core of Infinite Thread is composed of a polyester multistrand thread. More precisely, the core is made of polyethylene terephthalate. The coating and cogs of Infinite Thread are made of medical grade solid silicone. Similarly, Spring Thread comprises a polyester helix core together with soft medical‐grade silicone cogs. Spring Thread has more capacity to stretch. Besides, since the Infinite Thread has more notches, the tissue retention is higher. Table 1 summarizes the main features of both thread types.

**TABLE 1:** Main characteristics of the Infinite Thread and Spring Thread.

Feature	Infinite Thread	Spring Thread
Material	Polyester core with a silicone coating	Polyester core with a silicone coating
Elasticity	Nonelastic; maintains tension without elongation under body temperature or mechanical stress	Elastic; designed to recoil and provide lift
Needle and thread pairing	Provided separately from the threads	Provided anchored to the threads
Number of needles	5 different needles with varying bevels	Two flexible needles on both sides of the threads
Thread length	30 cm	30 cm
Needle length	19 cm	15 cm
Thread diameter	1.4 mm	0.5 mm
Number of notches	4 notches per 1.5 mm; 800 notches in total	24 notches per cm; 720 notches in total

To the best of our knowledge, the variation of the thread thickness has not been studied via ultrasonographic imaging. Our aim in this study was to evaluate this change in thread thickness according to the time after the procedure with these two types of silicone threads.

## 2 Materials and Methods

In this study, we included 51 patients who applied to a private dermatology clinic and underwent thread lifting. We included patients over 18 years of age, who had not previously undergone thread lifting, who were not pregnant or lactating, and who did not have any dermatologic disease. Informed consent forms were obtained from all patients. A minimum of 2 and a maximum of 12 threads were inserted in 51 patients included in the study. The entry point was above the zygomatic arch; one half of each thread was directed superiorly towards the scalp and the other half was directed inferiorly to the midface. The patients were evaluated by ultrasound between 0 and 91 months in terms of the visibility of the threads, thickness of the threads, migration, if any, according to the cannula entry point, echogenicity changes, and possible complications in these patients. The thickness of the threads was measured at the center of the inferior half placed in the midface region.

**FIGURE 1:**
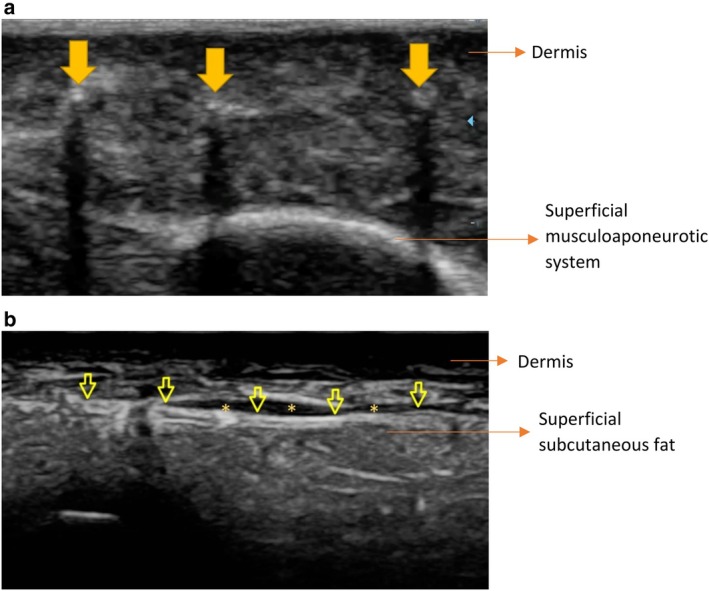
The ultrasonographic appearance of Infinite Thread placed in the malar region, 3 weeks after the procedure. (a) In the axial view, the threads are observed as echogenic structures that create acoustic shading posteriorly (thick arrows). (b) When the probe is placed in the same direction as the thread, the thread appears as a bilaterally echogenic line (yellow arrows). Note the fluid collection adjacent to the thread (asterisks).

All the ultrasound scans were performed by the first author, with Mindray DC‐80 X‐Insight; a high‐frequency (12–14 MHz) linear probe was utilized. When the probe was placed perpendicular to the direction of the threads, they appeared as circles with acoustic shading. When the probe was placed in the same direction as the threads, the Infinite Thread was observed as longitudinal lines with a hypoechoic center (Figure 1), whereas the Spring‐Thread had a hyperechoic center owing to the position of silicone.

The data obtained in the study were analyzed using SPSS (Statistical Package for the Social Sciences) for Windows 25.0. Descriptive statistical methods (number, percentage, mean, SD, etc.) were employed in evaluating the data. Independent *t*‐test was used to analyze variables with two groups, and one‐way analysis of variance (ANOVA) test was used to evaluate variables with more than two groups. To determine the normality of the data, skewness and kurtosis were examined, and since they were within the range of ±1.5, it was accepted that they showed a normal distribution.

The approval of İstanbul University‐Cerrahpaşa, Traditional and Complementary Medicine Application and Research Center Institutional Review Board was taken. The study was conducted in accordance with the principles of the Declaration of Helsinki. Informed consent was obtained from all the participants.

## 3 Results

**FIGURE 2:**
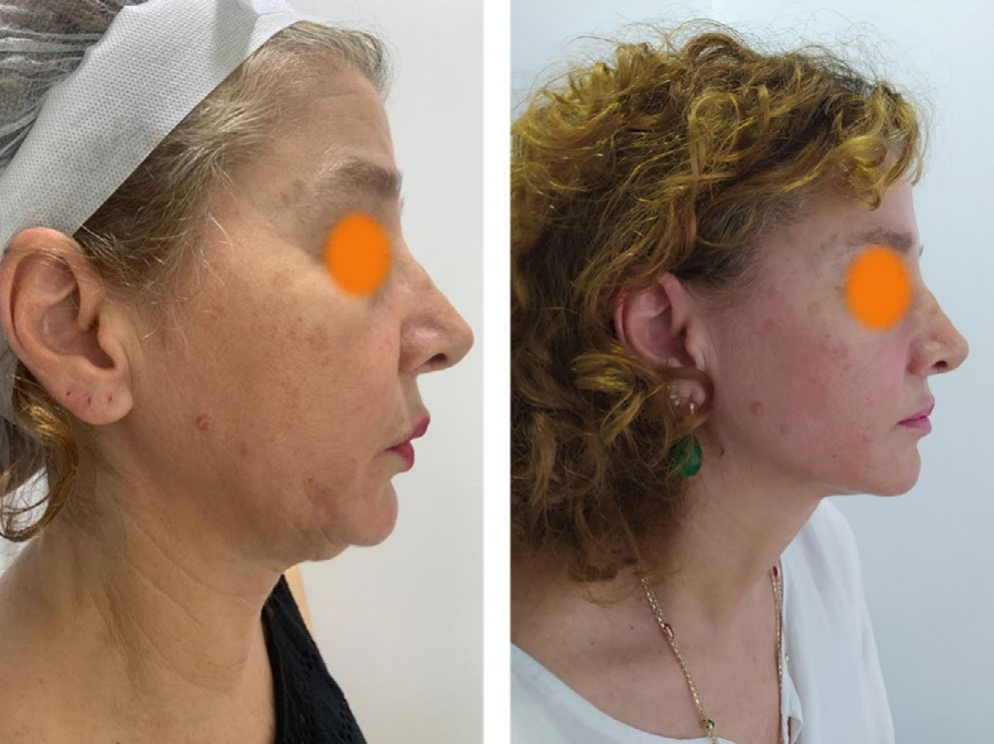
Photographs of a patient before and 3 years after thread lifting. (a) Before and (b) 3 years after. Informed consent was obtained from the patient.

**FIGURE 3:**
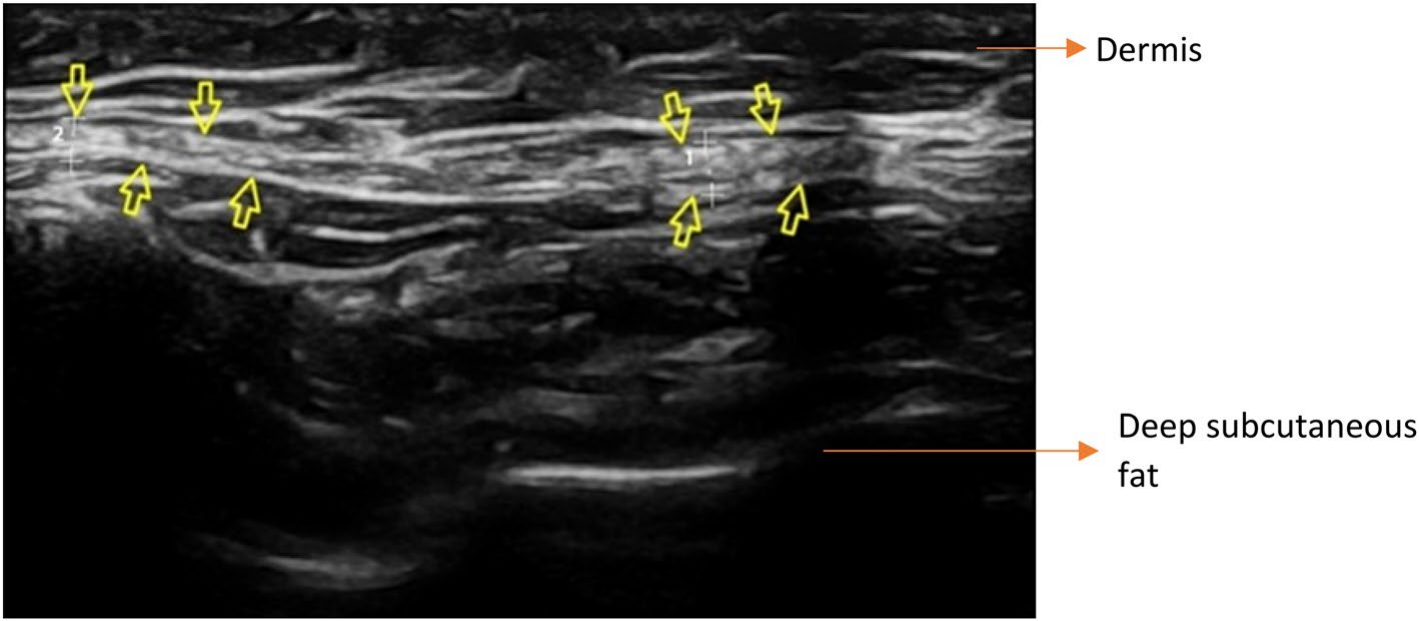
Ultrasound image of the same patient shown in Figure 2, taken 3 years post‐procedure. The echogenic linear structure indicated by the yellow arrows represents the silicone thread, embedded in the midface superficial subcutaneous fat. Note the consistent echogenicity along the entire course of the thread, without signs of fragmentation or surrounding inflammatory changes.

A total of 51 individuals participated in this study. The highest number of participants, 48–58 years old, accounted for 41.2%. Among the participants, 29 individuals (56.9%) underwent facial procedures. Twenty‐seven participants (52.9%) had 4 or fewer thread counts. Infinite Thread was used in a total of 41 individuals (80.4%). Twenty (39.2%) patients were measured after 6 months or less. The majority, comprising 21 individuals (41.2%), had a mean thread thickness between 1.37 and 1.41. Those with visible thread counts of 4 or fewer were the majority, consisting of 27 individuals (52.9%) (Table 2). The patient with the longest time after procedure had a measurement done 91 months after the procedure. The most common complication after the procedure was swelling. There was no migration in any of the patients, nor any prolonged adverse effect. We present the photographs and the ultrasonographic image of one of our patients as an example, in Figures 2 and 3, respectively.

**TABLE 2:** Summary of the patient and thread characteristics.

Variables		*n*	%
Age	47 or less	19	37.3
	48–58	21	41.2
	59 or more	11	21.6
Location	Face	29	56.9
	Neck	22	43.1
Thread count	4 or less	27	52.9
	5–6	14	27.5
	7 or more	10	19.6
Brand	Infinite Thread	41	80.4
	Spring Thread	10	19.6
Time after procedure	6 months or less	20	39.2
	7–24 months	13	25.5
	25 months or more	18	35.3
Mean thread thickness	1.37–1.41	21	41.2
	1.42–1.49	15	29.4
	1.50–1.53	15	29.4
Visible threads	4 or less	27	52.9
	5 or more	24	47.1

Table 3 presents the results of the independent samples *t*‐test conducted between mean thread thickness and anatomical location, and also between mean thread thickness and visible thread count. It was found that the mean thread thickness did not change statistically significantly according to the thread count (*p* > 0.05). It was observed that the thread thickness differed statistically significantly depending on the anatomical area where the procedure was performed (*t*(49) = 2.253; *p* < 0.05). It was observed that the mean thickness of the threads placed on the face was greater than that of those placed on the neck.

**TABLE 3:** Comparison of mean thread thickness according to anatomical location and visible thread count.

Mean thread thickness		*n*	Mean	SD	*t*	*p*
Location	Face	29	1.46	0.06	2.253	0.029*
	Neck	22	1.43	0.05		
Visible threads	4 or less	27	1.44	0.05	−0.869	0.389
	5 or more	24	1.45	0.06		

* *p* < 0.05 independent samples *t*‐test.

Table 4 shows the one‐way ANOVA results for the comparison of mean thread thickness among age, thread count, and time after procedure groups. It was observed that there was no significant difference in mean thread thickness among different age and thread count groups (*p* > 0.05). A statistically significant difference was detected in mean thread thickness among individuals with different times after the procedure (*p* < 0.05). The group who had their ultrasonography done 6 months or less after the procedure had a mean thread thickness lower than those who had 7–24 months and those who had 25 months or more after the procedure.

**TABLE 4:** Comparison of mean thread thickness according to age, thread count, and time after procedure.

Mean thread thickness		*n*	Mean	SD	*F*	*p*	Significant difference
Age	47 or less	19	1.45	0.05	0.892	0.417	
	48–58	21	1.45	0.06			
	59 or more	11	1.43	0.05			
Thread count	4 or less	27	1.44	0.05	0.639	0.532	
	5–6	14	1.45	0.05			
	7 or more	10	1.46	0.06			
Time after procedure	1. 6 months or less	20	1.39	0.01	63.230	0.000*	1 < 2
	2. 7–24 months	13	1.46	0.04			1 < 3
	3. 25 months or more	18	1.50	0.03			

* *p* < 0.05 one‐way ANOVA.

**FIGURE 4:**
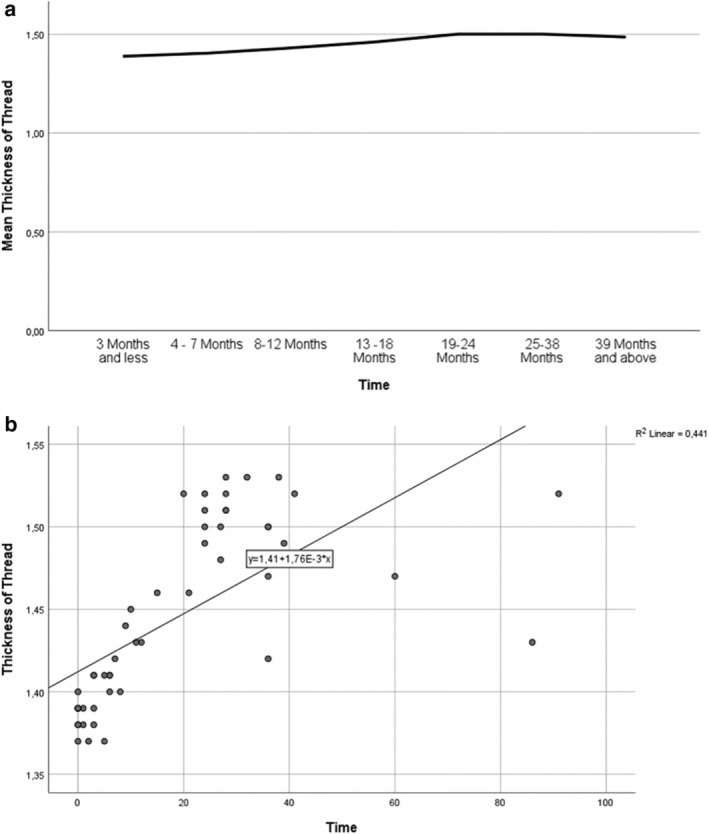
Graphs showing the variation of mean thread thickness with time. (a) The thread thickness increases until 25 months, then it stays constant. (b) Random dispersion of observation points around the regression line.

Figure 4 shows the variation of mean thread thickness with time. In Figure 4b, the data points appear scattered randomly around the regression line, which means there is no clear pattern or trend. This suggests that thread thickness does not consistently increase or decrease over time. The *R* ^2^ value—a measure of how well time explains the changes in thread thickness—was also low. This indicates that the amount of time since the procedure does not predict how thick the threads are. The thickness tends to stay within a stable range regardless of how much time has passed.

To assess the post hoc power of the study, we performed a power analysis using the “G. Power‐3.1.9.7” program. In the analysis of variance study concerning the relationship between time and mean thread thickness, *η* ^2^ = 0.725 was used as the effect size, and the alpha value was set to 0.05. In the post hoc analysis conducted with three groups and a total of 51 people, the power of the study was found to be 0.99.

## 4 Discussion

Since the early 20th century, cosmetic surgeons have been performing rhytidectomy procedures to reverse the facial signs of aging [9]. Although effective, the surgical rhytidectomy techniques may require prolonged operative time and convalescence, lengthy skin incisions, extensive dissection of the soft tissue, and thus may lead to numerous adverse effects [10]. Due to the risks of invasive methods, thread lifting, which is a minimally invasive procedure first introduced in the late 1990s, rapidly gained popularity [11]. For the time being, although the thread lifting procedure is widely used, objective studies concerning the durability of the threads are scarce. Furthermore, studies concerning the long‐term consequences of thread lifting did not utilize imaging for quantitative measurements.

**FIGURE 5:**
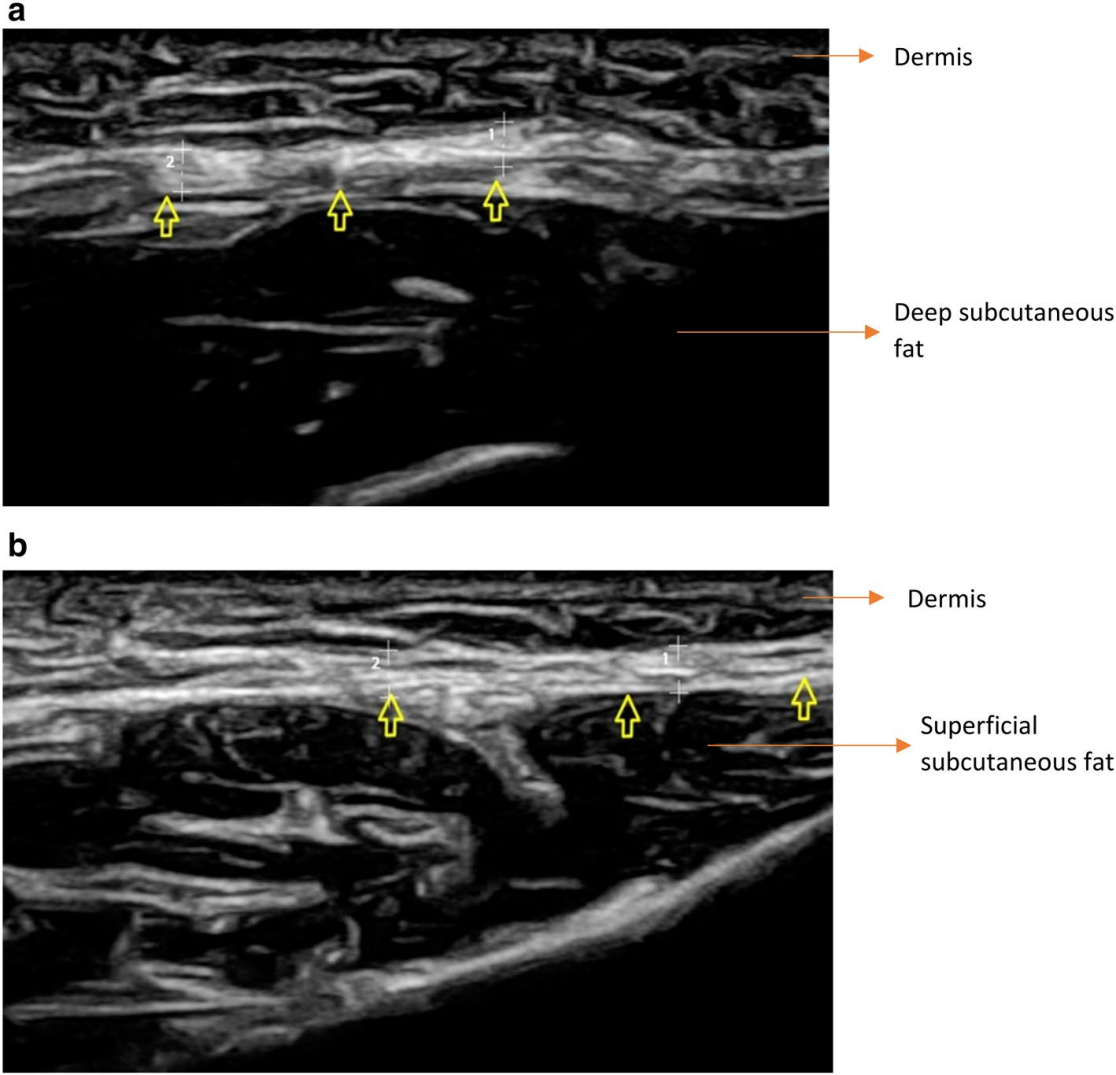
Two sonograms obtained from the midface of a case that was followed up for more than 7 years (86 months). Two different threads are shown in (a, b) indicated by yellow arrows. No changes in the placement of the threads were detected. A significant increase in thread thickness and echogenicity is clearly prominent to the eye.

To our knowledge, this is the first study in the literature to assess the changes in thread thickness by means of ultrasonography, according to the time after procedure. We had performed thread lifting using Spring Thread and Infinite Thread. The thickness of the threads was measured with ultrasonography after a certain time. It can be observed from Figure 4a that the thread thickness increases until 25 months, and then it stays constant. Although a statistically significant difference in thread thickness was observed between certain time groups, the variation was minimal and did not follow a consistent trend over time. The mean thickness remained within a narrow range, and regression analysis showed a low *R* ^2^ value, indicating no meaningful linear change. Clinically, this suggests that the threads maintain their structural stability for extended periods, even up to 91 months post‐procedure. The stable thread thickness may correlate with the absence of complications such as migration or loss of lifting effect, supporting the long‐term biocompatibility and mechanical integrity of the material. Previous studies on long‐term efficacy reported results based on patient satisfaction, surgeon's observation, and photographic evaluation [12]. For instance, Lycka et al. had followed up 117 patients for a period of 12–24 months; the patients had maintained 70% of their initial correction as determined by blinded photographic assessment [13]. Moreover, Lee et al. included 35 patients and used patient satisfaction with photography to evaluate the effects during 12 months; overall, 94.3% of the patients were satisfied [14]. All these evaluations are difficult to quantify, and they have their own bias. On the other hand, our study showed that the thread thickness did not change in the long span, which implies the high stability of the threads. One of our patients had her measurements done after 86 months and another after 91 months; the threads were still visible after 7 years (Figure 5).

The long‐term consistency in thread thickness observed in our study may be explained by foreign body reactions such as fibrosis and encapsulation, likely contributing to the mechanical stability of the thread in situ. These tissue responses may reinforce the positioning of the thread, reducing the likelihood of degradation, thinning, or migration.

There are conflicting results in the literature regarding the relationship between thread count and patient satisfaction. One study showed no relationship [15]; yet another study showed increased satisfaction with increasing numbers of threads [16]. Regardless of patient satisfaction, our analysis regarding the thread counts and their measured thickness rendered a *p* value of 0.532, meaning that there is no statistically significant relationship between thread count and thread thickness. Another variable that may affect the durability of the threads is the age of the individual. Nevertheless, our analysis pertaining to the age and the thread thickness had a *p* value of 0.417, which concludes that these two variables are not related to each other.

In addition to the face, thread lifting can also be used in the neck or other body parts such as the gluteal region or the abdomen [17, 18]. Comparable to the literature, in most of our patients (56.9%), thread lifting was applied to the face, and the rest of the patients underwent a neck procedure. It was notable that the analysis conducted for the relationship between the anatomical location and the thread thickness revealed a *p* value of 0.029. The thickness of the threads in the face was greater than those in the neck.

According to a recent systematic review by Pham et al., the most common transient adverse effects associated with thread lifting are swelling, ecchymosis, erythema, bleeding, and hematoma formation. Facial asymmetry is the most common prolonged side effect [11]. Similar to the previous studies, the most common adverse effect that we encountered was swelling. The patients who underwent ultrasonography during the first month after the procedure revealed fluid collection in the images, which was interpreted as a serohemorrhagic fluid (Figure 1). All the adverse effects that we detected were transient and self‐resolving. None of the patients reported a prolonged or a serious adverse effect.

Our study has several limitations. Particularly, its retrospective design limits our ability to control confounding variables and introduces the possibility of selection bias. The patient population was not randomized or stratified by thread type, anatomical region, or other clinical variables. Additionally, the small sample size, especially within subgroups such as neck procedures or longer follow‐up durations, limits the statistical power for some comparisons. Because the study relied on available ultrasonographic data, not all patients had standardized imaging intervals, which may affect comparisons over time. These factors should be considered when interpreting the generalizability of our findings. Future prospective studies with more balanced cohorts and standardized imaging follow‐ups are needed to validate and expand upon our results.

## 5 Conclusions

Thread lifting is a safe and effective method to reverse the impact of gravity and aging, such as the sagging of the midface or deepening of the nasolabial folds. Previous studies had investigated the durability of the threads in terms of qualitative measures. In this ultrasonographic study, we documented the stability of the threads by showing their constant thickness over time. Nevertheless, our study is limited considering the design and the small sample size. Further studies with larger patient populations and prospective trials are required to confirm our conclusions.

## Author Contributions

Hüray Hügül performed all the thread‐lifting and ultrasonography procedures. Kadir Atacan Yıldız and Rozerin Neval Altunkalem wrote the original draft. Züleyha Aydın performed the statistical analysis. Zekayi Kutlubay contributed to the conceptualization and methodology of the article. All the authors reviewed and approved the final version of the manuscript.

## Ethics Statement

The authors confirm that the ethical policies of the journal, as noted on the journal's author guidelines page, have been adhered to and the appropriate ethical review committee approval has been received. The study was reviewed and approved by İstanbul University‐Cerrahpaşa, Traditional and Complementary Medicine Application and Research Center IRB.

## Consent

Consent for the publication of recognizable patient photographs was obtained by the authors. All patients gave consent with the understanding that this information may be publicly available.

## Conflicts of Interest

The authors declare no conflicts of interest.

## Data Availability

The data that support the findings of this study are available from the corresponding author upon reasonable request.
